# Workflow for phenotyping sugar beet roots by automated evaluation of cell characteristics and tissue arrangement using digital image processing

**DOI:** 10.1186/s13007-023-01014-0

**Published:** 2023-03-31

**Authors:** Nelia Nause, Facundo R. Ispizua Yamati, Marion Seidel, Anne-Katrin Mahlein, Christa M. Hoffmann

**Affiliations:** 1 Institute of Sugar Beet Research, Holtenser Landstraße 77, 37079 Göttingen, Germany; 2 Bildungsakademie Der Universitätsmedizin Göttingen, Humboldtallee 11, 37073 Göttingen, Germany

**Keywords:** Sugar beet, Histology, Histologic evaluation, Digital image processing, Phenotyping

## Abstract

**Background:**

Cell characteristics, including cell type, size, shape, packing, cell-to-cell-adhesion, intercellular space, and cell wall thickness, influence the physical characteristics of plant tissues. Genotypic differences were found concerning damage susceptibility related to beet texture for sugar beet (*Beta vulgaris*). Sugar beet storage roots are characterized by heterogeneous tissue with several cambium rings surrounded by small-celled vascular tissue and big-celled sugar-storing parenchyma between the rings. This study presents a procedure for phenotyping heterogeneous tissues like beetroots by imaging.

**Results:**

Ten *Beta* genotypes (nine sugar beet and one fodder beet) were included to establish a pipeline for the automated histologic evaluation of cell characteristics and tissue arrangement using digital image processing written in the programming language R. The identification of cells has been validated by comparison with manual cell identification. Cells are reliably discriminated from intercellular spaces, and cells with similar morphological features are assigned to biological tissue types.

**Conclusions:**

Genotypic differences in cell diameter and cell arrangement can straightforwardly be phenotyped by the presented workflow. The presented routine can further identify genotypic differences in cell diameter and cell arrangement during early growth stages and between sugar storage capabilities.

**Supplementary Information:**

The online version contains supplementary material available at 10.1186/s13007-023-01014-0.

## Background

Imaging is a valuable data acquisition tool to evaluate spatial relations between anatomical structures [1]. Furthermore, microscope imaging techniques and the introduction of machine learning techniques in cell biology have enabled plant phenotyping at the cellular level [2]. Sugar beet plants are remarkably interesting objects for automated cell phenotyping because of its heterogeneous storage root tissue structure. Sugar beets comprise several cambium rings surrounded by small-celled vascular tissue and big-celled sugar-storing parenchyma between the rings [3]. The vascular tissue is more robust in tension than the parenchyma, and the rings are denser in the outer parts of the root, especially near the periderm [4, 5].

For sugar beets, considerable genotypic differences in tissue strength have been detected, particularly in the puncture resistance of the periderm and the firmness of the underlying tissue [6, 7]. These differences influence damage susceptibility during harvest, pathogen infestation during storage, and sugar losses. However, little is known about tissue structure and cell characteristics influencing tissue strength so far.

Genotypic differences in the number and distance of the cambium rings have been described between sugar beet and fodder beet [8, 9]. *Beta Vulgaris* cultivars can be divided into four cultivar groups: Leaf Beet, Garden Beet, Fodder Beet and Sugar Beet [10]. These groups differ in stored sugar content, and therefore a large difference in the type, size, and number of cells [11]. Genetic differences in cell size are determined very early during plant development [12, 13], which could be helpful for the screening of genotypes in the context of breeding. So far, cell sizes must primarily be determined manually. Automated cell features extraction like size, shape, and wall thickness on microscopic images were run for different tissue types of a single vascular bundle in a sugar beet root after automatically clustering cells from individual morphological features [14]. However, studies of cell sizes considering different genotypes and larger areas of the root storage containing different tissue types and several cambium rings have not yet been carried out.

For plant breeding, fast and objective methods for quantifying anatomical features are indispensable. In other sciences like medicine, cell counting, and classification is of high relevance, too, and is applied in high throughput scenarios [15, 16]. However, to our knowledge, these methods are optimized to specific tissues showing distinct cell characteristics. The challenge in the use case beet root is the heterogeneity of cell characteristics belonging to the same tissue type: e.g., a cell of the storage parenchyma of an outer ring has different characteristics than a cell of storage parenchyma which is closer to the center of the beet root. This requires to take into account the location of the individual cells within the tissue and is also the prerequisite to identify and count cambium rings. Moreover, the identification of intercellular spaces is not possible by size or shape. We are not aware of any example from medicine or other disciplines which are comparable to this setting. Therefore, this study aimed to develop and compile a method for the automated evaluation of histological images by digital image processing for phenotyping of *Beta* genotypes. Here, we provide an image analysis workflow, which (i) differentiates cells from intercellular spaces and image artifacts such as dirt, damaged plant tissues, or air bubbles, (ii) determines the number and position of cambium rings in sugar beet roots, (iii) identifies different tissue types by subsequent clustering of morphologically similar cells, and (iv) extracts a set of morphometric data. The morphological data can then be used to distinguish the differences between the genotypes concerning their yield formation processes and their storability. The workflow was coded in R, a widely used free and open-source programing language for scientific analysis, thus helping to integrate the image evaluation into statistical evaluation processes and to transfer this method to other laboratories, to this effect the code of the worflow and an example image can be found at the additional file section [1, 2].

## Results

**Fig. 1:**
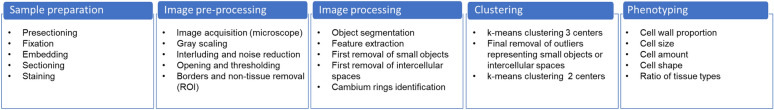
Schematic overview of subsequent steps from sample preparation to phenotyping. The process involves sample preparation, image preprocessing, image processing, clustering, and phenotyping

A schematic overview of all steps from sample preparation to phenotyping is presented in Fig. 1, where the different steps can be seen grouped according to their stage. First, the steps related to the preparation of the samples are mentioned, followed by the processing and analysis of the images. Afterwards the image clustering to finally obtain the phenotypic information is presented (Additional file 2).

### Sample preparation

For each of the ten genotypes, four cuboids from individual beet roots were embedded in paraffin. At least three sections per beet root were used for staining and one representative section was chosen for image acquisition and further processing as described in material and methods.

### Preprocessing of images and definition of the region of interest (ROI) by identification of non-tissue containing areas and objects touching the edges of the image

**Fig. 2:**
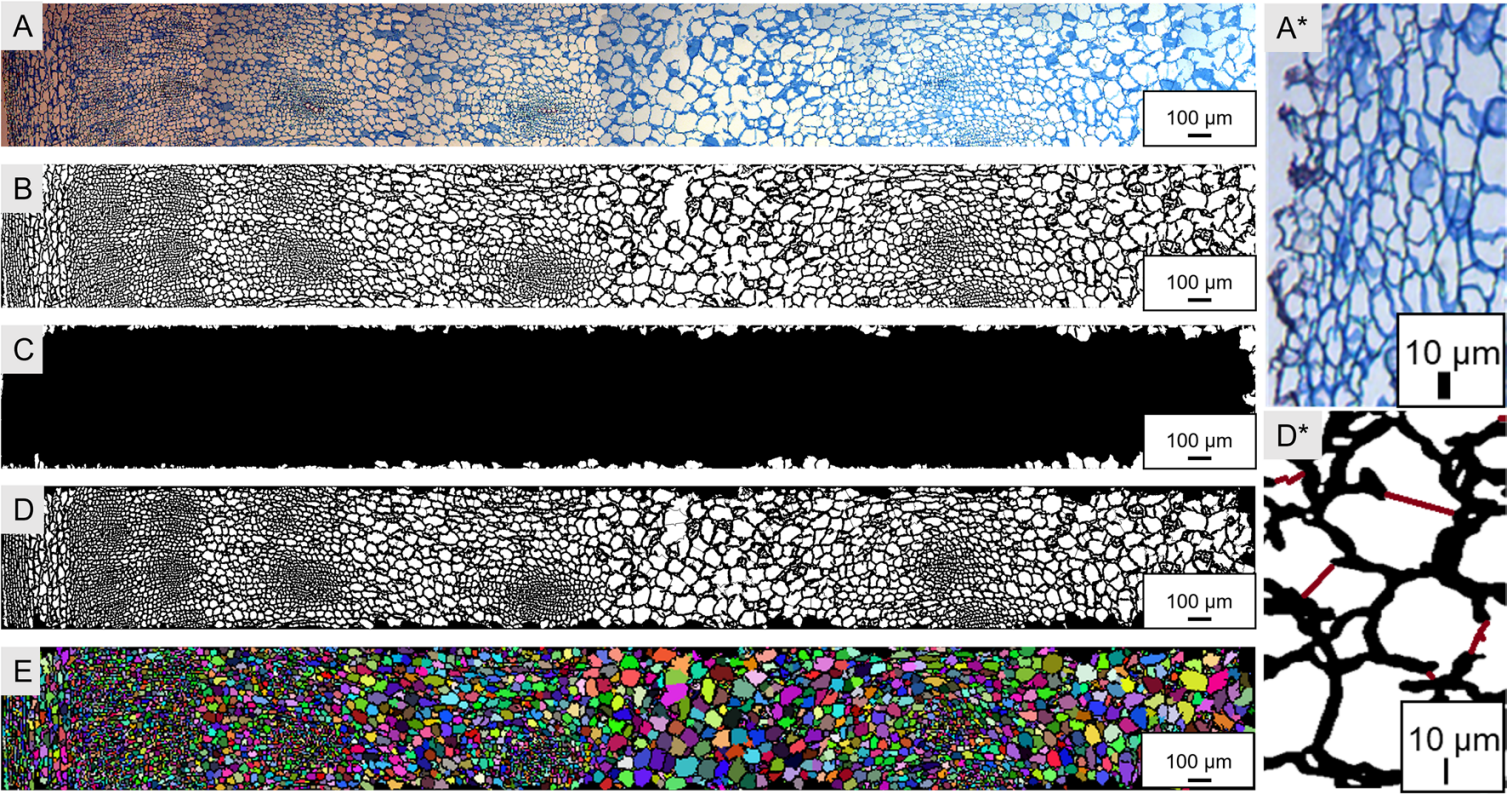
**A**–**F** Subsequent stages of image processing. **A**: The starting image, stitched and aligned from several light microscopy images of fuchsin-chrysoidine-astra blue (FCA)-stained transverse section of paraffin-embedded storage root tissue of sugar beet. A*: sample of an irregular edge. **B**: the same image as in A after subsequent image preprocessing, including thresholding (Otsu), opening, and hole filling. **C**: Cells touching the picture's edges are excluded from the analysis. **D**: After edge removal. D*: Magnified example of a damaged cellular wall enclosure area (red lines represent the artificially closed area). **E**: Individual objects are identified by watershed segmentation and are labeled by different colors

As a preprocessing step, every original image (Fig. 2A) was converted to grayscale. Furthermore, interluding, noise reduction, and opening were carried out as recommended and described in [17]. However, at the filtering and opening steps, the parameters of each function were slightly adapted to our images, using a value of sigma = 2 for the standard deviation of the Gaussian filter and by selecting the brush shape = disc, with size 18 and 21 respectively for the first and second opening cycle. Also, automatic thresholding (Otsu method, [18]) was applied. At this point, the images were converted to black and white (Fig. 2B).

### Elimination of irregular edge of the peridermal area

Due to the rounded shape of the periderm, each image shows areas of the image that could not contain any biological target tissue. For the correct functioning of the workflow, the periderm should be oriented towards the left edge of the image. Figure 2A* shows an example of an irregular edge. This area is prone to hold air bubbles and other materials that are potential artifacts. Therefore, it is necessary to mask and remove this area before starting the process inside the target tissue material.

For identification and removal of this area, only the first 5% from the left image border towards the right are considered to keep the workflow efficient. A high opening (erosion followed by dilatation; brush = 50) and hole filling identified the objects of interest. Holes are defined as areas of dark pixels surrounded by lighter pixels [19]. This area is removed from the processed black and white image.

### Removal of cells touching the edges of the image

At the edge of the images, incomplete cells can be seen because parts of the cells are lying outside of the photographic frame; those cells are considered as “edge-touching cells”. To assess cell sizes, it is necessary to consider only complete cells; therefore, partial cells touching the edge must be eliminated.

Objects whose pixel reach the edge of the image were identified using a process proposed by the Bioconductor project [20]. The process created a mask of the objects belonging to the border and another mask with the objects not belonging to the boundary. The number and morphological features of the identified edge-touching objects were documented. The information of the left edge was merged with the incomplete edge cells to perform the edge feature calculation (Fig. 2C).

The region of interest (ROI) is the area after removing non-tissue-containing sites and edge-touching objects from the original image. Finally, the ROI was reapplied to the grayscale image to continue processing (Fig. 2D).

### Closing damaged cells and final individual features extraction

Due to the delicate nature of cells, cells might be damaged during the preparation process of the tissue sections, and their surface becomes connected. The watershed algorithm allows cell walls to be rejoined. Therefore, it is necessary to generate a matrix with each pixel's distance inside the cells from the nearest cell wall pixel before determining the watershed. In our matrix, the cell interior is considered the foreground and the cell wall the background. The distance was computed with the distance map function (Distmap) in the Euclidean way.

The watershed algorithm segments the ROIs, and each object's morphological features were calculated. The area corresponding to the cell membranes and the site of the cell interior was determined in this step. In the tissue density image (Fig. 2D), high values (black) correspond to cell membranes, and low values (white) to the background. The amount of cell membrane per image was determined. With the function “ComputeFeatures” of the EBImage package, each segmented object's morphological information or features in the images were extracted into a table. This table consists of data (columns) for each detected object (lines or rows) in the image. The following information about the shape was recorded: area, perimeter, mean radius, the standard deviation of the mean radius, max radius, and min radius. The information about the object image moments contained the center of mass x, mass y, elliptical fit major axis, elliptical eccentricity, and object angle.

The diameter of each cell was calculated as 2*mean radius; the conversion from pixel to micrometer was calculated as diameter multiplied by the factor 1.015228, representing the pixel size for the image on the 10 × microscope used.

### Identification of number and position of cambium rings

**Fig. 3:**
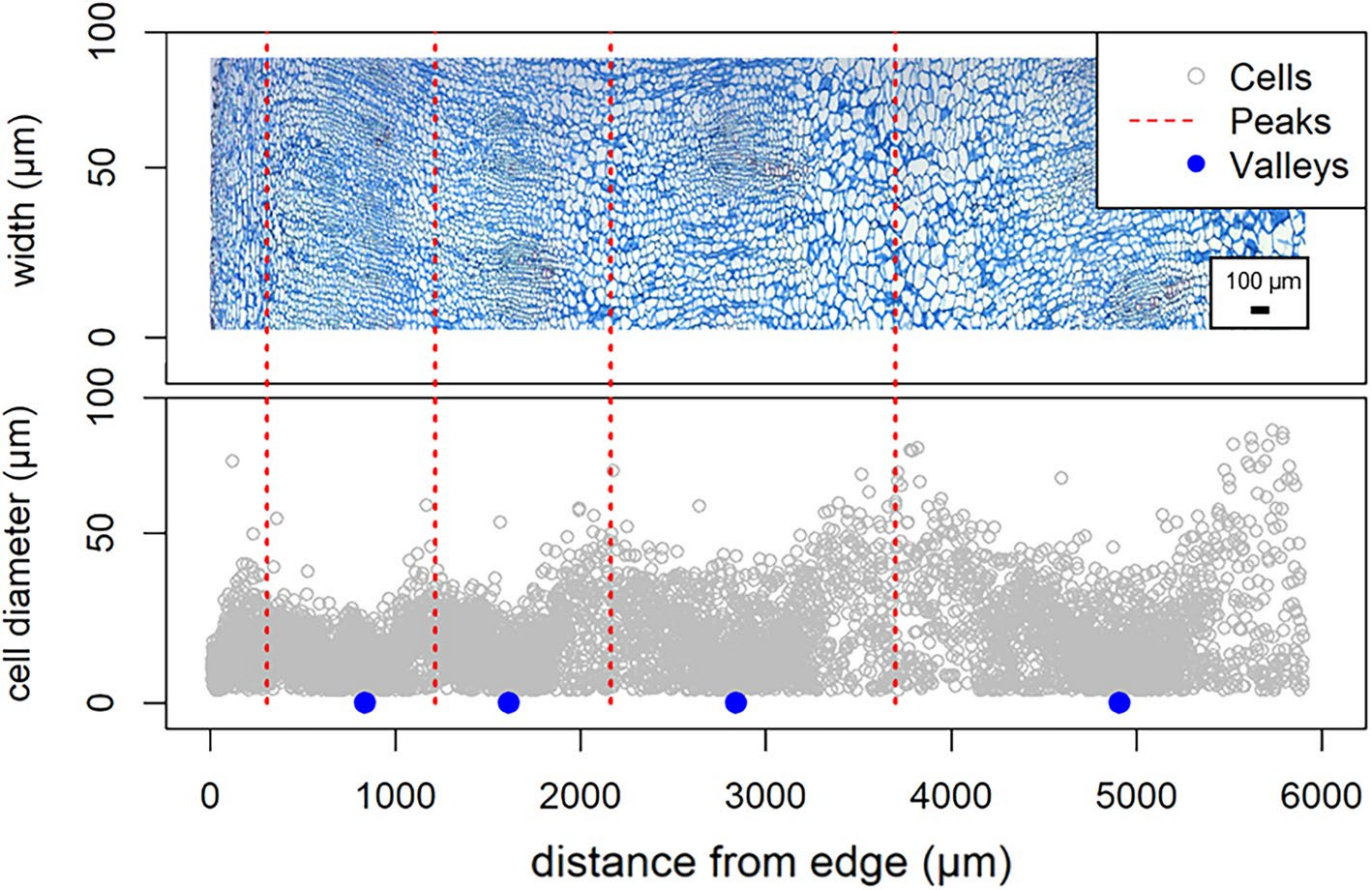
Detection of cambium rings and automatic rings spacing of sugar beet roots. The upper part shows a stitched and aligned image from several light microscopy image of fuchsin-chrysoidine-astra blue (FCA)-stained transverse section of paraffin-embedded storage root tissue of sugar beet. The histogram shows cell diameter as a function of distance from the left edge. The red dashed lines represent the cambium rings (peak) and the blue dots represent the center between two adjacent rings (valley)

The cell diameter and the distance of the cells from the left edge of the image is shown in Fig. 3. The beetroot tissue is composed of cells of different sizes. The maximal cell size increased from the outer part of the beet on the left towards the inner part on the right. The distribution of cell sizes along the x-axis represents the alternating occurrence of small-celled areas where the cambium rings are located and big-celled regions storing parenchyma. Using part of the code and algorithm suggested by [21] using the S3 Infrastructure for Regular and Irregular Time Series from ZOO package [22], the position of the peaks (storing parenchyma) and valleys (cambium rings) in the histogram were determined. Peaks were detected after the application of smoothing to find the local maximum (using as window width value of w = 150 and as a span argument for the loss function of span = 0.2). Valleys were detected using the same method after inversion of the y-axis. In addition to the position, the number of cambium rings was also computed by numbering them from left (outermost ring) to the right starting with 1.

Detected valleys and peaks are listed and numbered from the left edge inwards. Thus, it is possible to classify the cells between which the number of valleys or peaks is found. This information was added to the table obtained by ComputedFeature. This additional information allows the differentiation of cells into smaller groups during clustering. For example, there are usually relatively small cells in areas close to the epidermis. In this area, the presence of a cell of a slightly more significant size than the surrounding cells will not be identified as different if this is analyzed and compared with the cells of the whole sample; however, when determining clusters between the cambium rings, that distinct cell will be identified.

### Distinction between real cells, intercellular spaces, and artifacts

After segmentation, several objects did not necessarily represent cells. This was due to biological reasons like voids occurring between adjacent cells (intercellular spaces) as well as artifacts generated due to the nature of the image itself or the filtering process by over-segmentation. For the identification of as many of these non-real objects or spaces as possible and their subsequent elimination from the analysis, different strategies were used, as explained below.

**Fig. 4:**
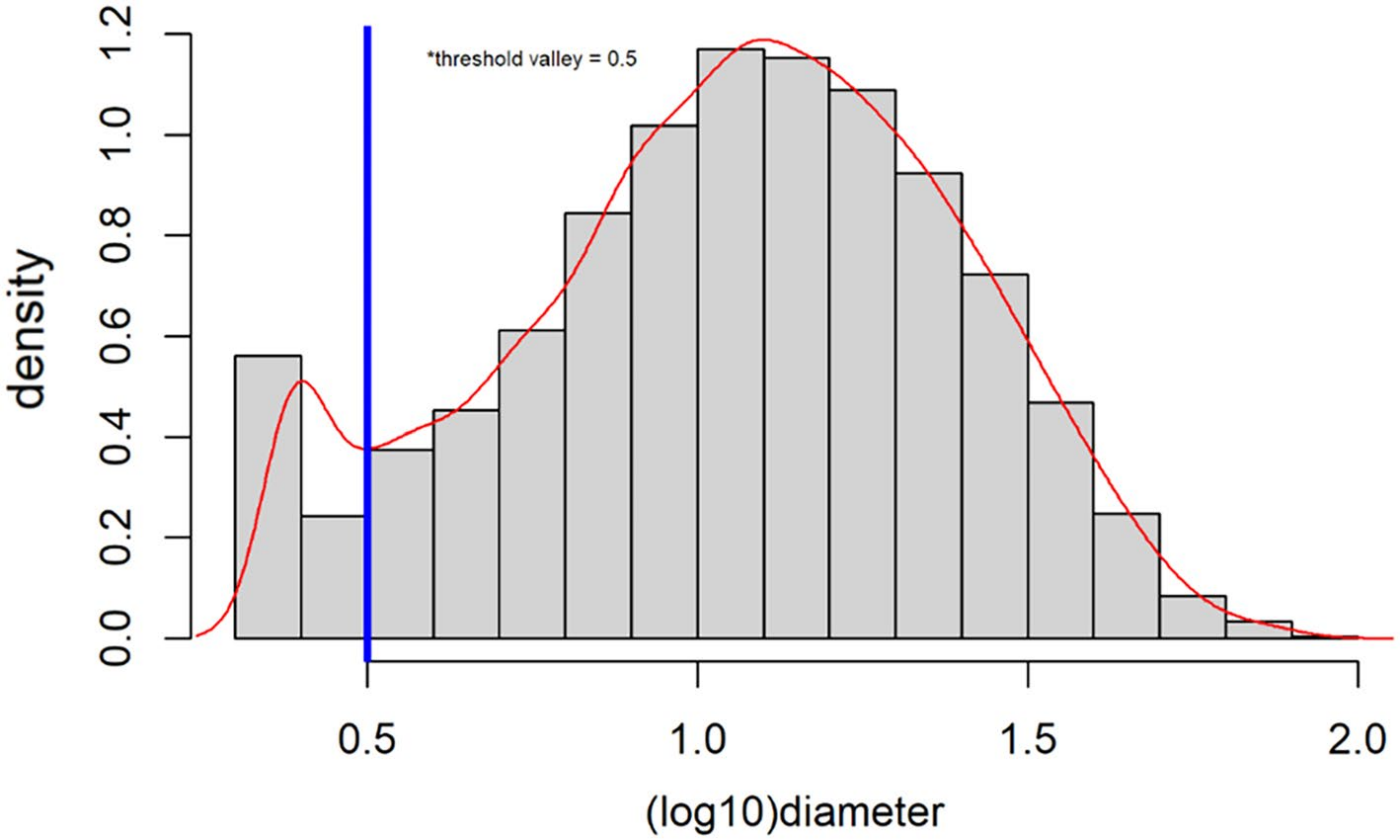
Histogram of cell diameter (log10) to determine the threshold for removing small objects. Bars represent the cell frequency for each 0.1 µm distance from the left border of the slide, the red line represents the continuous derived distribution, and the blue line is the automatic threshold delimitation of the two peaks of the curve (valley)

First, all objects of small size were dropped for the differentiation of cells from non-cellular objects. To accomplish this, the threshold valleys function of the benmack/threshold package was used. First, a frequency histogram of the size of the objects was determined based on the log_10_ (diameter) of the features (Fig. 4). If the frequency deviates from a normal distribution by showing two peaks, the benmack/threshold package determines the intermediate point between the two peaks. This point is the valley of the curve and the newly determined cut-off point to drop the lower outlier that escapes the normal distribution. Objects with a log_10_ (diameter) below the threshold value were eliminated. If no threshold point was found, a value of 0.5 was used, standing for a diameter of 3 µm. If valleys were located above 0.8, 0.8 was considered the new value threshold to avoid dropping viable cells.

**Fig. 5:**
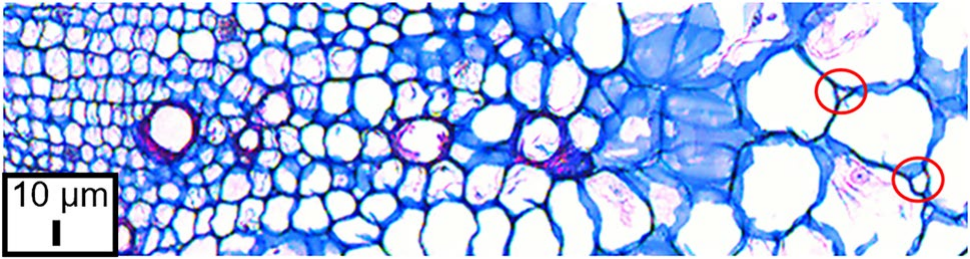
Light microscopy image of fuchsin-chrysoidine-astra blue (FCA)-stained transverse section of paraffin-embedded storage root tissue of sugar beet. Lignified parts appear red, and non-lignified segments are blue. Red circles indicate intercellular spaces of different shapes

The remaining objects can either be cells or intercellular spaces. Careful microscopic examination of the tissue revealed that the intercellular spaces mainly occur between large cells (Fig. 5). They might be bigger than small cells in other tissue areas, and they can have quite different shapes. However, the intercellular spaces of beetroots are always smaller than the surrounding cells.

For each object a subsequent analysis of the neighboring objects was performed in a two-step-process to identify the intercellular spaces. First, outlier detection analysis was conducted based only on the diameter parameter of neighboring cells. Therefore, based on the x and y coordinates of the objects, the p nearest neighbors were identified by the k-dimensional tree (nn2 function of the RANN package). The maximum number of nearest neighbors to compute was set as default to k = 6. Based on the distance of the neighboring objects to the object of interest, quantiles (Q) and Interquartile Range (IQR) were calculated. Objects with a gap above Q2 + 1.5 * IQR were not considered as direct neighbors of the object of interest. Quantiles and IQRs were calculated for log_10_ of the diameter of the object of interest and its immediate neighbors. Objects with a log_10_ (diameter) below the Q1—1.5 * IQR were considered as outlier and probable intercellular space. They were removed from the dataset, while all remaining objects were initially defined as cells.

**Fig. 6:**
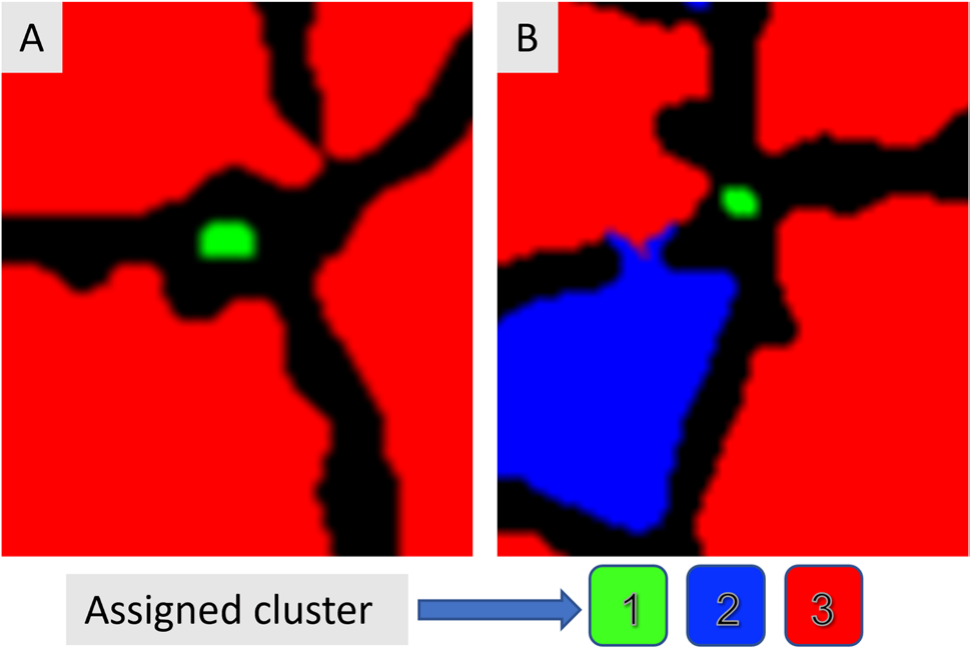
Cluster assignment. **A**: intercellular space classified as cluster 1 and surrounded by cells of cluster 3. **B**: intercellular space classified as cluster 1 and surrounded by different clusters. Cells classified as cluster 1 are marked in green, cluster 2 in blue, and cluster 3 in red. Cell walls are marked in black

The second, most resource-demanding strategy, is the clustering of the cells based not only on the diameter, but on all the morphological features obtained with “ComputedFeature” (except for data concerning the cell´s position; x and y, and the information about the ring number each cell is assigned to). Based on the possibility that there may remain 3 possible types of objects, respectively large cells, small cells and intercellular spaces, a clustering with 3 clusters was performed. Before clustering, the cambium rings and their cells belonging to each zone were determined and numbered. All input data was centered and scaled with the R base function “scale”, followed by a k-means clustering from the Stats package was used with a random set of 25 (nstart), maximum iteration of 1000 (iter.max), and 3 different clusters (centers). Since the cluster value assignment (1, 2 or 3) in this function is random, a reclassification is needed to achieve that different images always receive the same order of cluster values. It was determined that the smallest cells should continuously be assigned a value of 1, and the largest cells should always be assigned a value of 3. After obtaining the clusters, the cells were repositioned in the matrix, and the neighboring cells were analyzed again, using the Kd-tree, with the difference that, in this case, the neighborhood of the different types of clusters was analyzed. Considering that when the cells surrounding the cell under analysis are large, and this analyzed cell is small, the "cell" is considered as intercellular space. The number of cells that can surround an intercellular space is variable, and many possibilities and combinations are found. Therefore, two different criteria were used for the identification of intercellular spaces: (1) Small objects, assigned to cluster 1 surrounded by big cells assigned to cluster 3 (Fig. 6A). (2) Small objects, assigned to cluster 1 surrounded by a majority of cells assigned as cluster 3 but some cells as cluster 2, for instance, three as cluster 3 and one as cluster 2 as displayed in Fig. 6B. The decision if the central cell is an intercellular space requires the definition of "majority". Therefore, the following parameters are required: Number of surrounding cells, sum (SUM) and mean (MEAN) of the assigned cluster values of the surrounding cells; and the possible maximum sum (MAX) that could be obtained if all surrounding cells were assigned to cluster 3 (number of surrounding cells * 3). If SUM is greater or equal than MAX minus MEAN, the central cell can be considered an intercellular space and be deleted from the dataset. The entire table is continuously analyzed until no outlier is found.

### Final clustering and tissue discrimination

**Fig. 7:**
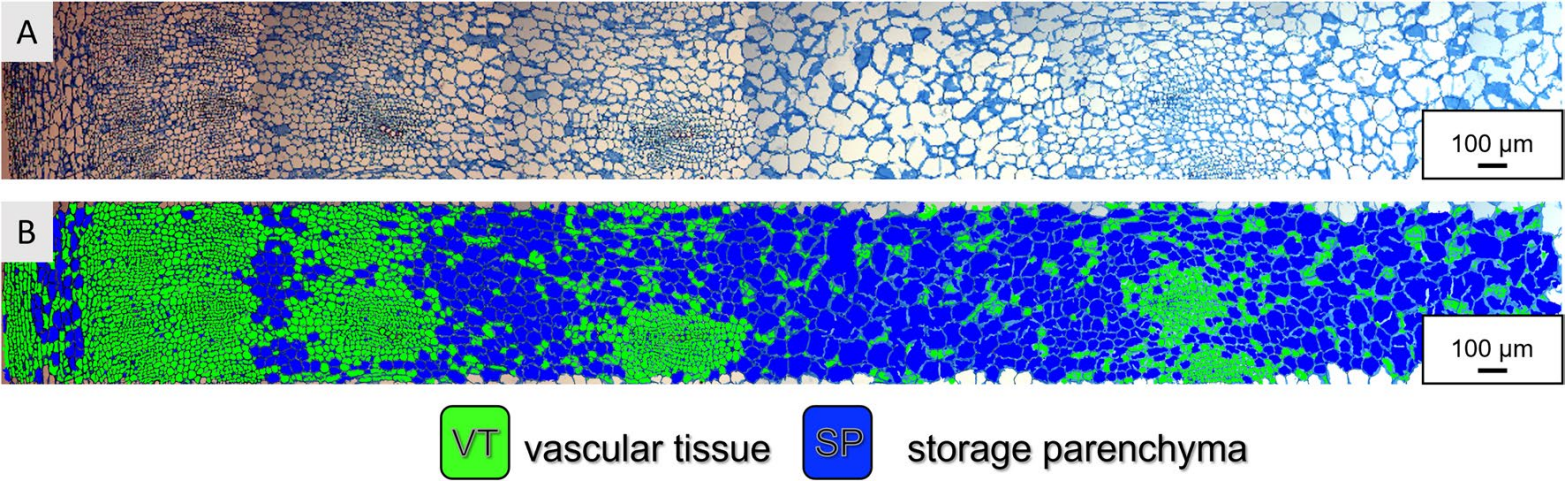
First 6 mm from the periderm (left side) towards the center of sugar beet root tissue (right side). **A**: stitched and aligned image from several light microscopy images of fuchsin-chrysoidine-astra blue (FCA)-stained transverse section of paraffin-embedded storage root tissue of sugar beet. **B**: Two groups of cells were identified, mainly reflecting the vascular tissue (green) and the storage parenchyma (blue)

After removal of the identified intercellular spaces, in theoretical perspective, two groups of cells remain in the dataset, the small and the large cells, representing the two main tissue types of beet roots, vascular tissue and storage parenchyma. Therefore, a new k-means clustering with the remaining data was performed, with a random set of 25 and maximum iteration of 1000, but this time with two clusters. As during 3-cluster clustering, the cluster number assignment is random, and a reclassification based on size is required, always assigning 1 to the smallest and 2 to the largest cells. The two classes of cells identified by digital image processing are marked in distinct colors in Fig. 7. The two clusters discriminate the two biological tissue types, as cluster one (green) mainly holds vascular cells, while cluster two (blue) mainly holds big cells of the storage parenchyma. To facilitate understanding and differentiate the cluster names with the 3-cluster clustering, small cells reclassified with cluster 1 will be assigned the letters VT for **v**ascular **t**issue and cluster 2 will be assigned the letters SP for **s**torage **p**arenchyma.

### Gathering and storing data

After completion of the clustering process, a table with the generated data was created; additionally, the name of the image file and extra information, such as the amount of the cell walls, were recorded in the new table. The amount of cell walls in the images (variable name: cellwallcount) was determined as the sum of all black pixels within the ROI of the black and white image, thus representing all pixels within the ROI excluding the cytoplasm and intercellular spaces. Afterward, the whole process was restarted with the next image in the list of images to analyze. The tables generated for each image are compiled into a final master table that contains all the accumulated information of all the images contained in the image list (Table 1).

**Table 1:** Variables contained in the final master table of the digital image analysis

From computefeature		From preprocessing, processing, and clustering	
*Variable name*	*Description*	*Variable name*	*Description*
s.area	Area size	Meandiameterµm	Diameter average calculated in µm
s.perimeter	Perimeter	Diameter_log	log10 of diameter
s.radius.mean	Mean radius	Q1	First quantile
s.radius.sd	Standard deviation of the mean radius	Q3	Third quantile
s.radius.max	Max radius	IQR	Interquartile range
s.radius.min	Min radius	nmbpeak	Number of peaks
m.cx	Center of mass x	nmdeep	Number of valleys
m.cy	Center of mass y	Cluster_drei	Classification of three clusters
m.majoraxis	Elliptical fit major axis	Cluster_zw	Classification of two clusters
m.eccentricity	Elliptical eccentricity	Imagetotalpix	Total image pixels
m.theta	Object angle	Innerpixarea	Total pixel ROI
order	Original order of cell sequence	Cellwallcount	Total pixel of cell wall
		Intercellcount	Total pixel of cell spaces
		Dataname	Image name

The final master table comprises all relevant data generated at different points in the process. Morphometric variables were measured by the ComputeFeature function. These variables were used to calculate further variables during preprocessing, processing, and clustering. For each variable, the name and a description were included

### Validation

The correlation between manual cell counting (Ground truth) and automated counting of sugar beet root cells (objects after watershed segmentation and final cell count) is illustrated in Table 2. In the set of reference images (one image for each of the ten genotypes), 15100 cells were identified in total by manual identification (ground truth), while 16790 were identified by automated image processing (final cell count). The coefficient of determination was 0.98.

**Table 2:** Validation of cell identification by comparison of the number of cells in ten microscopic images of sugar beet roots using manual counting

Genotype	Objects after watershed segmentation	Final cell count	Objects removed (%)	Ground truth
1	2688	2615	3	2562
2	1722	1517	12	1586
3	610	450	26	295
4	755	571	24	322
5	3203	2990	7	2911
6	2075	1914	8	1454
7	3488	3332	4	3106
8	1282	1138	11	1047
9	1850	1641	11	1403
10	737	622	16	414
Correlation to ground truth (R^2^)	0.9798	0.9815		

The applicability of the final clustering with two clusters was also assessed by expert interpretation of the groups regarding the known histology of the sample. It was confirmed that the clusters detected as one and two during clustering represented VT and SP, respectively.

### Phenotyping of different *Beta* genotypes

The first 6 mm from the periderm of ten *Beta* genotypes, nine sugar beet and one fodder beet, have been phenotyped based on the final data table created within the pipeline. An average of 4081 (genotype 10) to 7259 cells (genotype 7) were identified from the four samples per genotype. The maximal cell diameter in fodder beet was 129 ± 14 µm, and for sugar beet, it ranged from 72 ± 19 µm in genotype 9 to 112 ± 23 µm in genotype 4. The mean cell diameter was highest in fodder beet (21.8 µm, genotype 10). In sugar beet, it ranged from 16.6 µm (genotype 7) to 19.5 µm (genotype 3). The number of cambium rings in the 6 mm root tissue ranged from 4.5 (genotype 1) to 5.75 (genotypes 2, 3, and 5). Statistically, significant differences occurred for the number of cells and the mean cell diameter, but not for the number of cambium rings (Table 3).

**Table 3:** Cell characteristics of the distal 6 mm of root tissue of ten *Beta* genotypes; 1–9: sugar beet, 10: fodder beet

Genotypes	Number of cells	Max cell diameter [µm]	Mean cell diameter [µm]	Number of cambium rings	Amount of cell wall material [% of ROI pixels]
1	6046 ± 747^ab^	82 ± 6^ab^	18.3 ± 1.1^ab^	4.5 ± 0.5^a^	0.35 ± 0.0076^ab^
2	6036 ± 1326^ab^	87 ± 13^ab^	18.3 ± 2^ab^	5.75 ± 1.2^a^	0.35 ± 0.0140^ab^
3	5319 ± 513^ab^	90 ± 10^bc^	19.5 ± 1.11^ab^	5.75 ± 0.5^a^	0.35 ± 0.0097^ab^
4	5174 ± 243^ab^	112 ± 23^ab^	19.2 ± 0.62^ab^	5.5 ± 0.58^a^	0.35 ± 0.0066^ab^
5	6288 ± 986^ab^	90 ± 11^ab^	17.7 ± 1.67^ab^	5.75 ± 0.96^a^	0.36 ± 0.0169^a^
6	6949 ± 1777^ab^	81 ± 19^ab^	17.0 ± 2.38^b^	5.25 ± 1.2^a^	0.37 ± 0.0162^a^
7	7259 ± 433^a^	76 ± 14^c^	16.6 ± 0.68^b^	5.25 ± 1.2^a^	0.36 ± 0.0037^a^
8	5844 ± 1405^ab^	88 ± 23^ab^	18.6 ± 2.28^ab^	6.0 ± 1.6^a^	0.35 ± 0.023^ab^
9	7052 ± 1649^a^	72 ± 19^c^	16.8 ± 2.44^b^	6.25 ± 0.9^a^	0.37 ± 0.0213^a^
10	4081 ± 998^b^	129 ± 14^a^	21.8 ± 3.13^a^	5.5 ± 1^a^	0.32 ± 0.0201^b^
MSD	2713	31.8	4.6	2.5	0.037

Automatic determination from microscopic images of cell tissue with four samples per genotype. Mean and SD of the four images per genotype are indicated. MSD: minimum significant difference calculated by HSD test, alpha = 0.05. Different letters indicate significant differences between genotypes (p<0.01)

The mean cell diameter was significantly negatively correlated to the number of cells per genotype (R^2^ = 0.98, p ≤ 0.01) and to the amount of cell wall material identified in the microscopic images (R^2^ = 0.96, p ≤ 0.01; data not shown).

**Fig. 8:**
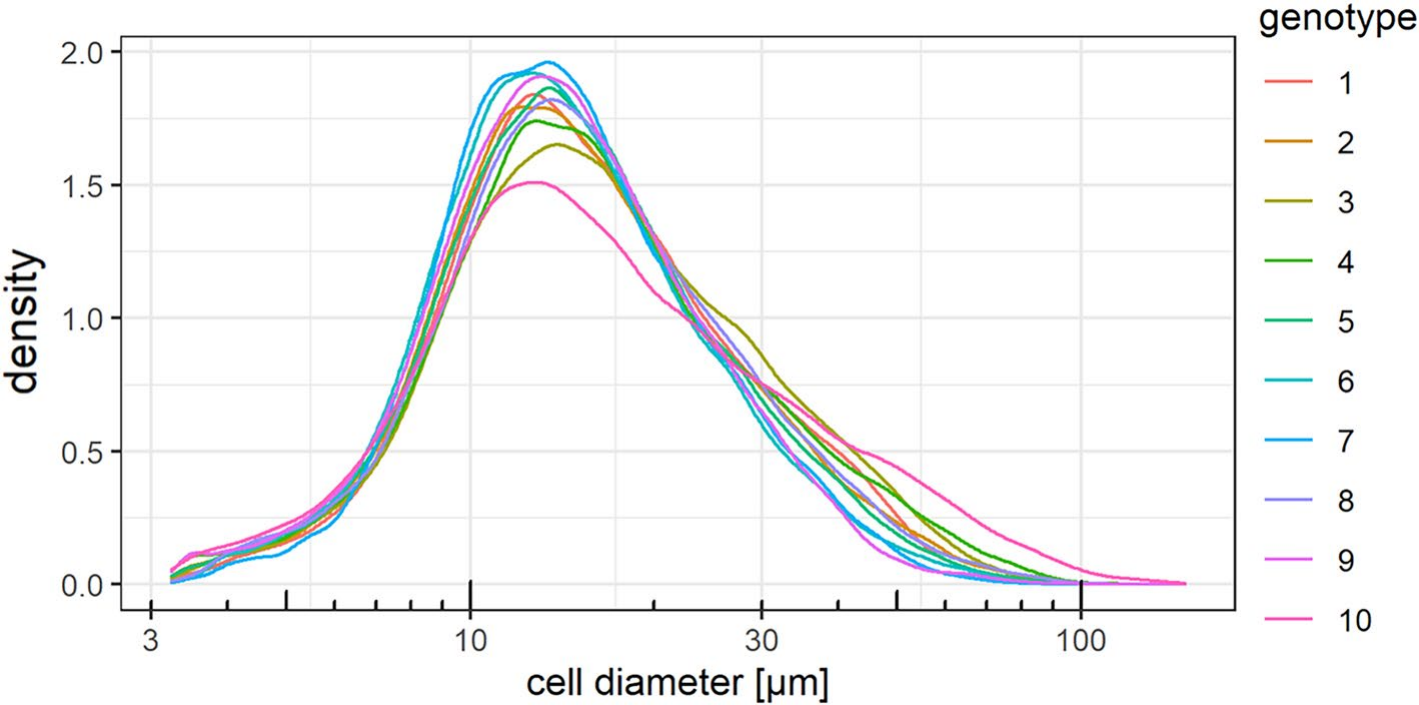
Kernel density estimate of the cell size distribution in the distal 6 mm of root tissue of ten *Beta* genotypes; 1–9: sugar beet, 10: fodder beet; four samples per genotype. The height of the curve is scaled such that the area under the curve equals one. The density estimate was performed with a Gaussian kernel and a bandwidth of 1

The distribution of cell sizes in the 6 mm distal root tissue per genotype is shown as Kernel density estimate in Fig. 8. All genotypes showed a peak at cells with a diameter of approximately 12 µm. The comparison between genotypes showed that this cell diameter had the highest abundance in genotypes 6, 7, and 9, and the lowest in genotypes 3, 4, and 10. At larger cell diameters, this relation changed, and genotypes 3, 4, and 10 had the highest abundance of cells with a diameter above 30 µm, and genotypes 6, 7, and 9 had the lowest.

**Fig. 9:**
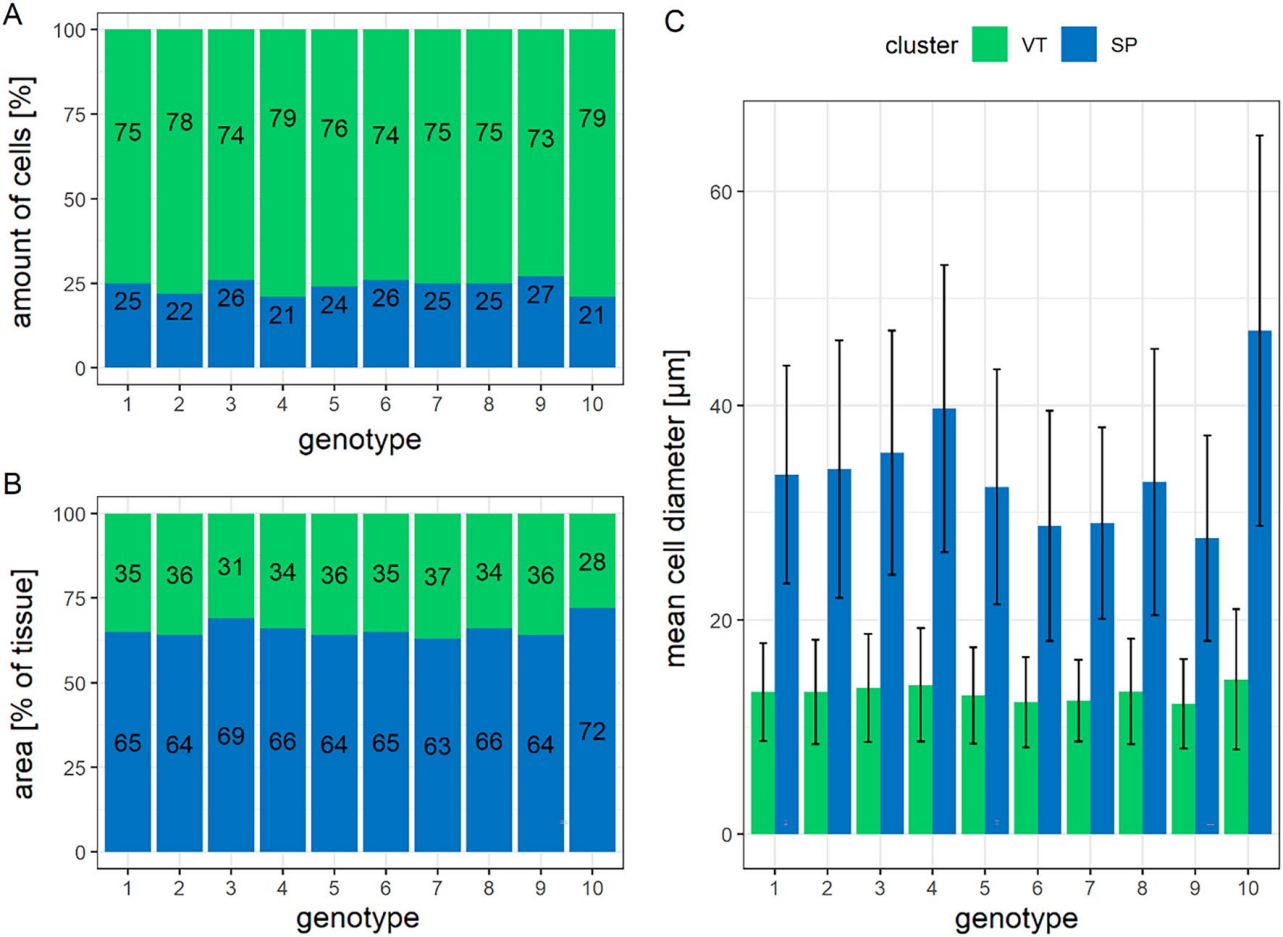
Allocation of the distal 6 mm of sugar beet root cells to clusters based on their morphological features. Genotype 1–9: sugar beet, 10: fodder beet. Cluster VT mainly represents vascular tissue; Cluster SP mainly represents storage parenchyma. **A**: percentage of cells per cluster, **B**: percentage area per cluster, **C**: mean cell diameter per cluster

For each genotype, most cells were assigned to cluster VT. Only a small variation between genotypes was found in the ratio of cluster VT to cluster SP, 73% (genotype 9) to 79% (genotypes 4 and 10) of the cells assigned to cluster VT (Fig. 9A). Due to the smaller cell sizes, however, cluster VT only took up 28% (genotype 10) to 37% (genotype 7) of the image area (Fig. 9B). The mean cell size was calculated separately for each cluster and genotype. Genotypic differences in cell size were more pronounced in cluster SP than in cluster VT (Fig. 9C). The ratio of cluster VT to cluster SP was relatively stable across genotypes (number of cells, 9 A, and tissue area, 9 B). Genotypic differences in cell diameter were more pronounced in the storage parenchyma (cluster SP; 9 C).

**Fig. 10:**
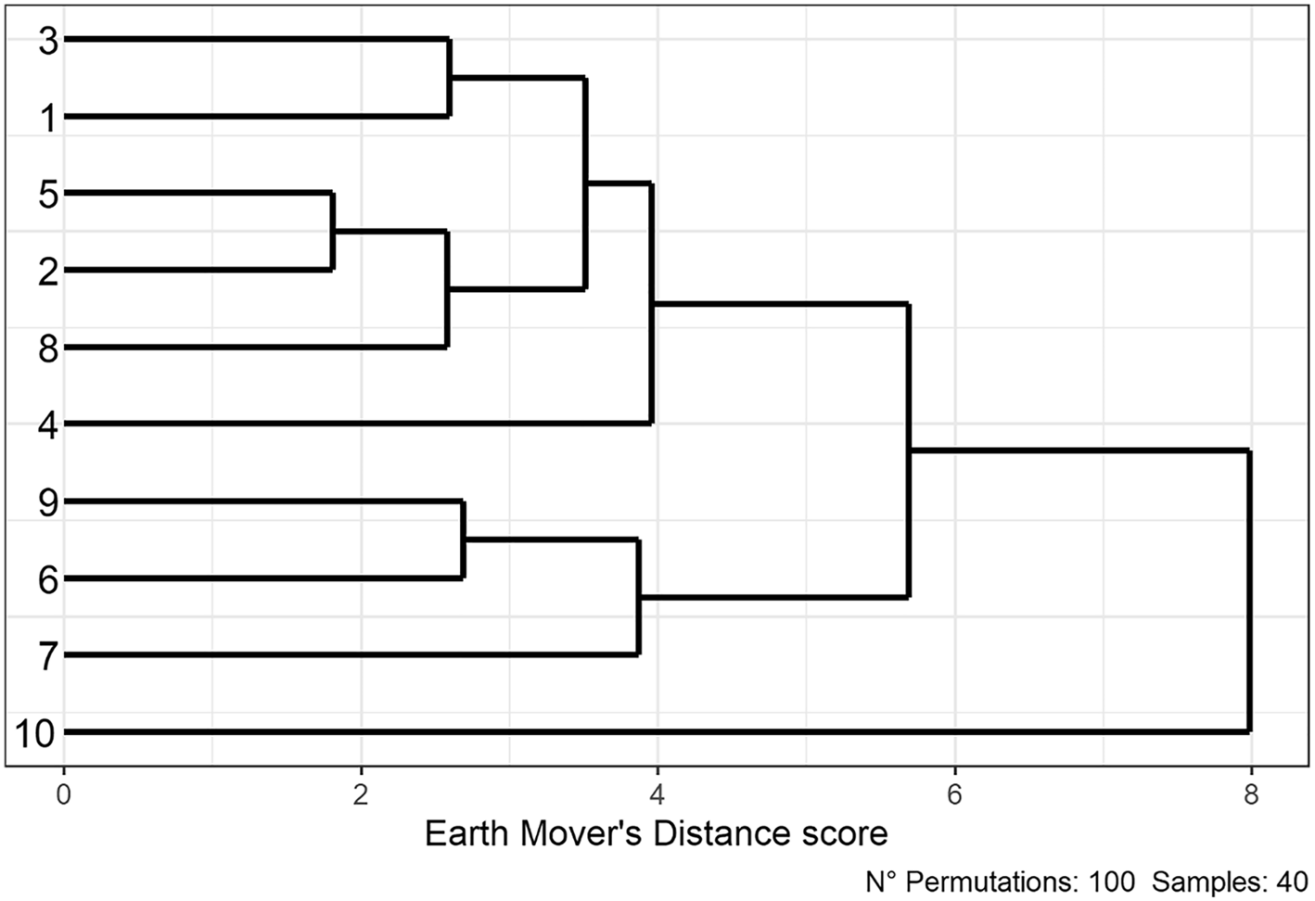
Dendrogram to visualize the similarity based on Earth Mover's Distance between *Beta* genotypes after digital image analysis of the distal 6 mm of root tissue. Genotype 1–9: sugar beet, 10: fodder beet. The Distance score between different genotypes is represented on the x-axis, and the different genotypes are displayed on the y-axis. The number of randomly computed permuted scores = 100 and n = 40

For the similarity analysis of the genotypes in a dendrogram, the values of each image belonging to the final master table (Table 1), with exception of the centers of mass, Q1, Q3, IQR, file name and the original order of cell appearance, which were not considered for analysis. For the other variables, the mean, standard deviation, and maximum and minimum value were calculated. The distance-based dendrogram revealed two branches at a distance of 7.98, where genotype 10 (fodder beet), differs from the other nine sugar beet genotypes. For sugar beet, two large groups could be distinguished in which genotypes 9, 6, and 7 belong to one group, and the remaining genotypes to the other group (Fig. 10).

The image analysis by an automated digital workflow with other phenotypic characteristics facilitates the identification of relevant cell characteristics and allows an extended phenotyping, which enables breeders to select for low damage susceptibility of sugar beets during harvest and low sugar losses during storage. A comprehensive phenotyping of the ten genotypes is out of the scope of this work, but we have calculated the correlation between puncture resistance of the beet roots (as indicator for their storability) and mean cell area as an example. Even though puncture resistance was measured on representative beets of the same genotype, but not on the individual beet used for image analysis, the correlation coefficient (R^2^ = 0.606, p ≤ 0.01; data not shown) shows a clear correlation between cell size and sugar beet tissue strength.

## Discussion

This study aimed to develop and compile a method for the automated evaluation of histological images by digital image processing for phenotyping of *Beta* genotypes. The provided image analysis workflow overcomes various difficulties in differentiating cells from intercellular spaces and image artifacts, determining the number and position of cambium rings in sugar beet roots, and identifying different tissue types. The workflow was developed and optimized for histologically evaluating sugar beet tissue. However, it can also easily be adapted for other tissues (evaluated with other root tissues, data not shown). It should be noted that despite the high performance of the algorithms to differentiate cells, some intercellular spaces and cells may even be misclassified. Also, the closure of broken cells may be the source of some artifacts due to over- and under-closure. Many of these algorithm confusions are determined by the quality of the sample preprocessing and the quality of the camera on the microscope.

In the following, we will discuss some possibilities of adjustments of the workflow for further research goals or other tissue types.

For automatic cell identification, discrimination between cells and intercellular spaces is indispensable. Typically, the shape of intercellular spaces differs from that of cells since a cell in contact with a void exhibit’s convexity due to its internal pressure. Therefore, intercellular spaces are deflated due to the convex surface of the inflated neighboring cells. Convexity can be quantified by conventional object descriptors [23], but they are not necessarily sufficiently discriminating to reliably identify intercellular spaces [24]. Pieczywek et al. [25] used circularity and shape roughness for separating cells from intercellular spaces in apple tissue. Remarkably, the new approach of comparing the surrounding cells with the ROI enabled the integration of differently shaped intercellular spaces in the heterogeneous sugar beet tissue. The close correlation of the cell numbers derived by automated and manual counting shows the high precision of this type of automated cell identification.

In histological images, the cell wall thickness is frequently of interest. However, it is difficult to measure it accurately due to its small size and non-uniformity, being thinner in its middle area than around its edges. Moreover, some cell walls may be inclined to the cutting surface, and some cuttings may be right through plateau borders. As a result, measured wall thickness may be slightly larger than the actual thickness of cell walls. As shown by Chen et al. [26], it is possible to reduce the errors induced by the non-uniformity of cell wall thickness by taking measurements near the middle area of cell walls where thickness is relatively uniform, and the error induced by cell wall inclination by carefully selecting cell walls that seem vertical to the cutting surface while measuring. However, this procedure is not suitable for an automated evaluation of big datasets. Guillemin et al. [14] used 13 images for the analysis of one vascular bundle of beetroot. With this resolution, the cell wall thickness could be determined for each cell. Travis et al. [27] estimated cell wall thickness by a similar procedure after a watershed segmentation. This approach simplifies extracting cell wall thickness profiles by collecting all measured values through the distance function along the skeleton, which would reveal local thickness variations within the cell perimeter. To keep the workflow efficient, a lower image resolution than required for such attempts, was used. If required, a determination of the cell wall thickness could be integrated into our workflow. A much more straightforward approach, not requiring high-resolution images, was used by Cybulska et al. [28], who assessed a "cell wall fraction" as the ratio of the total length of the perimeters of all objects and the summarized area of objects. In the presented study, the amount of cell walls in the images was determined as the sum of all black pixels within the ROI of the black and white image, thus representing all pixels within the ROI excluding the cytoplasm and intercellular spaces.

Estimating cell sizes from microscopic images underlies three interrelated types of bias: 1. Small cells are less likely to be caught by the tissue slice than large cells, 2. The measured cross-sectional cell radius is likely smaller than the actual cell radius since the cross-section does not pass precisely through the center of the cell, and 3. The imaging software has a cut-off parameter that prevents the measurement of cells below the cut-off. As shown by Lenz et al. [29] correcting these types of biases is possible by estimating tissue cell size and type. So far, the underlying shape and the real cell size distribution is unknown for sugar beet roots. We have refrained from correcting the measured cell size, as in other published studies of sugar beet roots, no adjustment was made either for real cell size [5, 9, 12, 14, 30, 31].

The creation of tables containing all generated data simplifies the calculation of additional study-specific parameters and the data usability in subsequent statistical analysis. In this study, the data were used for phenotyping of different *Beta* genotypes. Between genotypes, even in the distal 6 mm of root tissue statistically significant differences were evaluated for the number of cells and the mean cell diameter. Different tissue types were identified by clustering. Cluster 1 mainly contains vascular cells, and cluster 2 mainly contains cells of the storage parenchyma. For vascular cells, there is no size reference yet. The cell sizes measured in the storage parenchyma were on the lower end of the range reported in other studies [9, 30, 31]. This was mainly influenced by the fact that in the presented workflow, only the first 6 mm from the periphery to the center were considered. This resulted in a lower mean cell diameter than for more centered tissue of the beetroot, or for a cross section of the entire tissue due to the fact that the cell size increases towards the center of the beetroot [3].

The relationship of cellular characteristics identified with the presented workflow and the mechanical tissue properties of sugar beet genotypes is concordant with descriptions by [6, 7].

Although it is known that at least sugar beet and fodder beet differ in their total number of cambium rings [8, 9]. In the outer 6 mm of beetroot tissue, no statistically significant differences were observed, neither between sugar beet and fodder beet nor between different sugar beet genotypes. Hence, for the analysis of the number of cambium rings, a larger area of tissue, probably ranging from the periderm to the center of the beetroot must be observed.

## Conclusion

Overall, the results describe a workflow which offers a substantial benefit for digital image processing, as it enables an automated evaluation of histological images of extremely heterogenous sugar beet root tissue. The findings of this study can be understood as a confirmation of measuring features of the images in a fully automated manner. Compared to manual analysis, information can be extracted more efficiently within a short time and without any subjective bias. Additional use cases include other plant tissue phenotypic analysis workflows, as this approach can be integrated effortless.

This is a promising approach to supplying quantitative information, which can be used in further statistical analysis for phenotyping of different *Beta* genotypes. algorithm delivers cell-intrinsic information that makes it possible to analyze differences in cell characteristics and arrangement between genotypes. This opens a broad spectrum of possibilities to improve phenotypic tissue characterization, which in case of *Beta* genotypes are also related to yield formation [3, 12]. A subsequent study must consider the potential contribution of the cellular characteristics identified with the presented workflow to mechanical tissue characteristics.

## Material and methods

### Beet samples

In 2020, nine sugar beet genotypes provided by SESVanderHave, Belgium, differing in sugar content and root yield (genotypes 1–9) and one fodder beet (genotype 10) were grown in a field trial at Sieboldshausen, Lower Saxony, Germany. All genotypes were cultivated in a randomized block design with four replicates. After harvest, the beet tissue was immediately prepared for microscopic analyses in October.

### Sample preparation and analyses

#### Microscopic analyses

The beetroots were pre-sectioned to cuboids with an edge length of approximately 1 × 1 × 2 cm, whereby an area of 1 × 1 cm holds the periderm of the beetroot, and the 2 cm were oriented towards the center. The cuboids were fixed in AFE (90% alcohol (ethanol, 96%), 5% formalin (37%), 5% acetic acid (100%)) until embedding, at least for 1 week. Subsequently, the tissue was transferred to 70% EtOH overnight, followed by increasing concentrations of isopropanol (70%, 80%, 90%, 100%; 1 day per concentration), xylene (100%; 72 h at RT, 48 h at 60 °C), xylene:Paraplast (Leica Biosystems, Richmond, IL, USA) [1:1 (v/v)] 24 h at 60 °C. The xylene:Paraplast mixture was replaced with pure Paraplast and incubated for 7d at 60 °C. Specimens were embedded in Paraplast. Blocks were cooled to 4 °C for unmounting and sectioning. 10 µm thick slices were sectioned on a sledge microtome. Sections were stretched in a water bath at 42 °C, transferred to glass slides, and dried at room temperature, at which they were stored until staining.

Tissue sections were deparaffinized with xylene and rehydrated by incubation with decreasing concentrations of EtOH (100%, 96%, 70%, 50%, 30%; 2 min each) and 3 × 3 min H_2_O. Sections were stained with Fuchsin-Crysoidin-Astral Blue (FCA or Etzold; Morphisto GmbH, 11742.00100) for 7 min and washed with H_2_O (3 × 2 min) and isopropanol (30 s). The stained sections were covered with Euparal (Carl Roth GmbH & Co. KG, 7356.1) and coverslips.

Brightfield images were acquired using a Zeiss microscope (Axio Scope.A1) with a 10 × magnification lens, a Moticam Pro camera (1024 × 1360 pixels per image), and the software Motic Images Plus (version 3.0) at a scale of 1.015228 µm/pixel. The acquisition of adjacent images was needed to observe a representative sample area. Mosaic images were reconstructed from 6–7 adjacent images using the Image Composite Editor (Microsoft, version 2.0.3) to attend a representative sample area. All mosaic images were cropped to equal size (5910 × 690 pixels, corresponding to approximately 6 × 0,7 mm) and saved in TIFF format in the same orientation, with the periderm of the beetroot touching the left margin of the image.

#### Data analysis

The R statistical computing environment [32] was used for data analysis. In particular, the following packages were used: The Image processing and analysis toolbox for R EBImage [33, 34], together with the support provided by the Bioconductor project [35], benmack/threshold for thresholding based on peak and valley of a histogram curve analysis [36], Fast Nearest Neighbour Search (RANN) [37] for the identification of the nearest neighbors, ZOO [22] for the identification of cambium rings, Stats for k-means clustering (part of R), and Agricolae [38] for HSD-test.

The automated digital image processing methods were developed in this study and are described in the *Results* section.

To calculate the similarity, we proceeded in the same way as described in [39], calculating the pairwise Earth Mover's Distance score and then a hierarchical clustering of the distances. With all these values, a dendrogram was designed to facilitate the visualization. As input values were used several values obtained at the end of the image analysis process. The parameters will be shown in more detail in the results section.

#### Manual cell counting for technical validation

To determine if the workflow is effective and comparable, manual cell counts were performed on a reference set of 10 original, non-stitched images (one per genotype), which were taken as ground truth. No pre-processing was done on the images. Using the software QGIS (version 3.20.1), each visually identified cell was labeled, and the total number of cells was determined. The same ten images were analyzed with automated image processing.

#### Measurement of puncture resistance of the beet root

The puncture resistance test was performed according to Kleuker and Hofmann [7] on five representative roots per replication with three measurements on each root using a texture analyzer (TA.XTplus100, Stable Micro Systems, Godalming, UK) with a puncture probe (diameter 2 mm) and a crosshead speed of 60 mm min^−1^. The means per root were summarized to a mean per replication. The Force_mean_ is the average force measured in the 5 mm underlying the periderm and describes the tissue firmness.

## Supplementary Information

**Additional file 1.** R Script for the automated evaluation of cell characteristics and tissue arrangement.

**Additional file 2.** An example of a stitched and aligned mosaic image from several light microscopy images of fuchsin-chrysoidine-astra blue (FCA)-stained transverse section of paraffin-embedded storage root tissue of sugar beet.

## Data Availability

The datasets used analyzed during the current study are available from the corresponding author on reasonable request.
