# Supplementary figures and images for: Workflow for phenotyping sugar beet roots by automated evaluation of cell characteristics and tissue arrangement using digital image processing
Source: Plant Methods. 2023 Mar 31;19:35. doi: 10.1186/s13007-023-01014-0 (PMC10064576; doi:10.1186/s13007-023-01014-0)

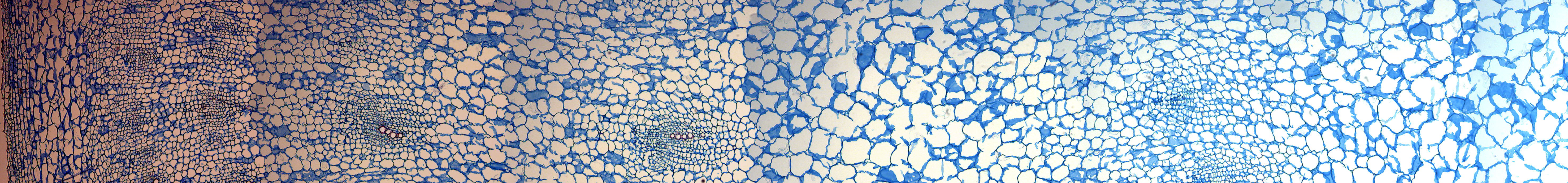

Supplement: Supplementary file 1 — Additional file 1. R Script for the automated evaluation of cell characteristics and tissue arrangement. [file 13007_2023_1014_MOESM1_ESM.tiff]
