# Supplementary material for: Workflow for phenotyping sugar beet roots by automated evaluation of cell characteristics and tissue arrangement using digital image processing
Source: Plant Methods. 2023 Mar 31;19:35. doi: 10.1186/s13007-023-01014-0 (PMC10064576; doi:10.1186/s13007-023-01014-0)
Supplement: Supplementary file 2 — Additional file 2. An example of a stitched and aligned mosaic image from several light microscopy images of fuchsin-chrysoidine-astra blue (FCA)-stained transverse section of paraffin-embedded storage root tissue of sugar beet. [file 13007_2023_1014_MOESM2_ESM.docx]

**Workflow for phenotyping sugar beet roots by automated evaluation of cell characteristics and tissue arrangement using digital image processing**

Nelia Nause^*1^, Facundo R. Ispizua Yamati ^*1^, Marion Seidel^2^, Anne-Katrin Mahlein^1^, Christa M. Hoffmann^1^

corresponding author: Facundo R. Ispizua Yamati, Ispizua@ifz-goettingen.de

^1^Institute of Sugar Beet Research, Holtenser Landstraße 77, 37079 Göttingen, Germany

^2^Bildungsakademie der Universitätsmedizin Göttingen, Humboldtallee 11, 37073 Göttingen, Germany

# **# libraries and packages**

library(EBImage)

library(RCurl)

library(ggplot2)

library(dplyr)

library(zoo)

library("threshold")

library(RANN)

library(ggstatsplot)

# **# necessary functions**

## # Function argmax for detection of the Cambium rings

argmax <- function(x, y, w=1, ...) {

n <- length(y)

y.smooth <- loess(y ~ x, ...)$fitted # smooth

y.max <- rollapply(zoo(y.smooth), 2*w+1, max, align="center")

delta <- y.max - y.smooth[-c(1:w, n+1-1:w)]

i.max <- which(delta <= 0) + w

list(x=x[i.max], i=i.max, y.hat=y.smooth)}

# **#directories for load and save**

dataset<-"D:/ Dataset" #select folder containing images

abb_2class<-"D: /graphs /class2/" # folder to save image with 2 clusters

abb_3class<-"D: /graphs/class3/" # folder to save image with 3 clusters

abb_valley<-"D:/ graphs/Valleys" # folder to save image with peaks and valleys

Table_raw<-"D:/Results" # folder to save results tables

setwd(dataset)

myFiles <- list.files(pattern="*tif")

**# start-parameters as default**

TH_bas<-0.5

#sigma every RGB Chanel

Sigma1<-2

Sigma2<-2

Sigma3<-2

brush1<-18

brush2<-21

opening<-3

neighbors_to_check=6

**# pipeline**

round<-1

for (filename in myFiles){ # loop that loops through all files in the selected folder

setwd(dataset)

cells <- readImage(filename) #read image

grayimage<-channel(cells,"gray") #convert to gray

cellsSmooth = Image(dim = dim(cells))

sigma = c(Sigma1, Sigma2, Sigma3)

for(i in seq_along(sigma)) {cellsSmooth[,,i] = filter2( cells[,,i],filter = makeBrush(size = brush1, shape = "gaussian",sigma = sigma[i]) )}

disc = makeBrush(brush2, "disc")

disc = disc / sum(disc)

nucMask = cellsSmooth[,,1] - filter2(cellsSmooth[,,1], disc) > 0

grayimage<-channel(nucMask,"gray")

thres <- grayimage > (otsu(grayimage)) #thresholding

## # opening

open.br<-makeBrush(opening, shape="disc", step=TRUE)

c2_thres_op <- opening(c2_thres, open.br)

c2_thres_op = fillHull(c2_thres_op)

## # bwlabel form gray to BW boolean 0 and 1

objects = bwlabel(c2_thres_op)

## # elimination of irregular border on left side

otsu_thres <- grayimage > (otsu(grayimage) ) # otsu thresholding

forbords = bwlabel(otsu_thres) # convert in black and white image (0-1)

forbords[150:nrow(forbords),]<-0 # inserts 0 in the whole image from column 150 onwards

open.br<-makeBrush(50, shape="disc", step=TRUE)

thres_op <- opening(forbords, open.br)

thres_op = fillHull(thres_op)

fordelete<-bwlabel(c2_thres_op)

objects[fordelete==1]<-1 # remove big border

nmask = watershed( distmap(objects),1)

## # subset for boundary pixels

objects = nmask

dims = dim(objects)

border = c(objects[1:dims[1],1], objects[1:dims[1],dims[2]],

objects[1,1:dims[2]], objects[dims[1],1:dims[2]])

# extract object identifiers at the boundary

ids = unique(border[which(border != 0)])

# create a mask containing objects at the boundary

boundary = as.vector(objects)

boundary[which(!(objects %in% ids))] = 0

imagetotalpix=dims[1]*dims[2]

innerpixarea<-sum(as.matrix(boundary) == 0L)

boundarypixarea<-sum(as.matrix(boundary) != 0L)

boundary = Image(boundary, dims)

rand<-bwlabel(boundary)

rand[fordelete==1]<-0

Bmask = watershed( distmap(bwlabel(rand)),1)

# display(colorLabels(Bmask), all=TRUE, method="raster")

B1 <- computeFeatures.shape(Bmask)

B2 <- computeFeatures.moment(Bmask)

features_boundary <- as.data.frame(cbind(B1, B1))

# create a mask containing all objects that do not intersect the edge of the image

inner = as.vector(objects)

inner[which(objects %in% ids)] = 0

inner = Image(inner, dims)

# segmentation

nmask = watershed( distmap(bwlabel(inner)),1)

allcells<-paintObjects(nmask,inner, col='black', thick=T, closed=T)

allcells<-channel(allcells,"gray")

allcells<-bwlabel(allcells)

cellwall<-!bwlabel(allcells+boundary)

cellwallcount<-sum(cellwall, na.rm = T)

# extract features

FS2 <- computeFeatures.shape(nmask)

FS3 <- computeFeatures.moment(nmask)

features<- as.data.frame(cbind(FS2, FS3))

features$order<-as.numeric(rownames(features))

original_list<-as.numeric(features$order)

features$meandiameterµm <- features$s.radius.mean*1.015228*2

features$diameter_log<-log10(features$meandiameterµm) # Logaritmus b 10

## # detection of values too small to be a cell and detection of a normal distribution of cell sizes

tryCatch( { TH_N <<- threshold_valleys(features$diameter_log)[1]}

, error = function(e) {TH_to_use<<-TH_bas}) # trycathc continue after error: sometimes the image already contains a good normal distribution and the valley detection does not find the point between the two valleys and presents an error. to avoid this, trycatch is used.

if (is.na(TH_N) || TH_N < TH_bas ) {TH_to_use<-TH_bas} else {

TH_to_use<-TH_N}

if (TH_to_use > 0.8 ) {TH_to_use<-0.8} #max limit for threshold valley

features<-features[which(!features$diameter_log <TH_to_use),]

# first outlier cleaning controlling the 6 neighboring cells of each cell

xy<-cbind(features$m.cx,features$m.cy)

closest <- nn2(data = xy, query = xy , k=6) ## Nearest Neighbour Search

for (valx in 1:nrow(features)) {

test_dist<-closest$nn.dists[valx,2:5]

iqr <- IQR(test_dist)

Q <- quantile(test_dist, probs=c(.25, .75), na.rm = FALSE)

above <- Q[2]+1.5*iqr

limit<-max(which(test_dist<above)+1)

cell_to_check<-features$diameter_log[closest$nn.idx[valx,1]]

atoi<-features$diameter_log[closest$nn.idx[valx,1:limit]]

iqr <- IQR(atoi)

Q <- quantile(atoi, probs=c(.25, .75), na.rm = FALSE)

low<- Q[1]-1.5*iqr

features$Q1[closest$nn.idx[valx,1]]<-Q[1]

features$Q3[closest$nn.idx[valx,1]]<-Q[2]

features$IQR[closest$nn.idx[valx,1]]<-iqr

if (cell_to_check>=low) { features$outlier[closest$nn.idx[valx,1]]<- 1} else {

features$outlier[closest$nn.idx[valx,1]]<- 0}

}

features<-features[which(features$outlier==1),]

features$outlier<-NULL

## # valley and peak detection after “meandiameterµm”

data_mean<-features %>%group_by(m.cx)%>%

summarise(meandiameterµm = median(meandiameterµm))

x<-data_mean$m.cx

y<-data_mean$meandiameterµm

peaks <- argmax(x, y, w=100, span=0.2)

features$nmbpeak<-1

valpeakmax<-length(peaks[1]$x)

valx=1

valpeak=1

for (xrow in 1:nrow(features)){

for (valpeak in 1:valpeakmax) {

if (features$m.cx[xrow]>=peaks[1]$x[valpeak]){features$nmbpeak[xrow]<-valpeak}}}

deeps<- argmax(x, 1/y, w=100, span=0.2) # now the Y-axis is inverted to determine the valleys or deeps

features$nmdeep<-1

valdeepmax<-length(deeps[1]$x)

valx=1

valdeep=1

for (xrow in 1:nrow(features)){

for (valdeep in 1:valdeepmax) {

if (features$m.cx[xrow]>=deeps[1]$x[valdeep]){features$nmdeep[xrow]<-valdeep}}}

setwd(abb_valley)

imgname=paste(gsub("\\..*","",filename),"_peaks_valleys.tiff",sep="")

tiff(imgname ,compression='lzw',width=22,height=12,units='cm',res=300,pointsize=8)

plot(features$m.cx, features$meandiameterµm, cex=0.75, col="Gray")

lines(x, peaks$y.hat*100, lwd=1) #$

y.min <- min(y)

sapply(peaks$i, function(i) lines(c(x[i],x[i]), c(y.min, peaks$y.hat[i]), col="Red", lty=2))

points(x[peaks$i], peaks$y.hat[peaks$i]*100, col="Red", pch=19, cex=1.25)

points(x[deeps$i], deeps$y.hat[deeps$i]*100, col="blue", pch=19, cex=1.25)

dev.off()

## # clustering

data_drei <- features

## # removal of all non-numerical and information that is not relevant or biasing for clustering

data_drei$m.cy<-NULL

data_drei$m.cx<-NULL

data_drei$order<-NULL

data_drei$outlier<-NULL

## # scaling

data.scaled <- as.data.frame(apply(data_drei, MARGIN = 2, FUN = scale))

data.scaled.clusters <- kmeans(data.scaled, centers =3, nstart = 25, iter.max = 10000) # nstart und interactionen

data.scaled$cluster <- data.scaled.clusters$cluster

## # definition of same cluster order for all images (starting with the lowest value for small cells)

features$Cluster_THREE <-as.factor(data.scaled$cluster)

cluster_1max<-mean(features$diameter_log[features$Cluster_THREE==1])

cluster_2max<-mean(features$diameter_log[features$Cluster_THREE==2])

cluster_3max<-mean(features$diameter_log[features$Cluster_THREE==3])

list_clust<-as.data.frame(rbind(cluster_1max,cluster_2max,cluster_3max))

list_clust$id<-c(1,2,3)

list_clust <-list_clust[with(list_clust, order(list_clust$V1)), ]

list_clust$new_id<-c(1,2,3)

features$Cluster_1_tochange<-features$Cluster_THREE

features$Cluster_2_tochange<-features$Cluster_THREE

features$Cluster_3_tochange<-features$Cluster_THREE

features$Cluster_THREE[features$Cluster_1_tochange==list_clust$id[1]]<-list_clust$new_id[1]

features$Cluster_THREE[features$Cluster_2_tochange==list_clust$id[2]]<-list_clust$new_id[2]

features$Cluster_THREE[features$Cluster_3_tochange==list_clust$id[3]]<-list_clust$new_id[3]

features$Cluster_1_tochange<-NULL

features$Cluster_2_tochange<-NULL

features$Cluster_3_tochange<-NULL

features$Cluster_THREE<-as.factor(features$Cluster_THREE)

features$Cluster_THREE <-as.numeric(features$Cluster_THREE)

test_out <-0

features$outlier<-NULL

features_rmb_inter<-features

## # analysis of number of neighboring objects (default=6) and their assigned cluster for each object. If an object is assigned to cluster 1 and all surrounding cells are greater than 2, this value is considered as intracellular space. This elimination process proceeds in a boucle until no further intercellular space is found.

k=6

while (test_out ==0) {

xy<-cbind(features_rmb_inter$m.cx,features_rmb_inter$m.cy)

closest <- nn2(data = xy, query = xy , k = neighbors_to_check)

valx=1

for (valx in 1:nrow(features_rmb_inter)) {

test_dist<-closest$nn.dists[valx,2:k]

iqr <- IQR(test_dist)

Q <- quantile(test_dist, probs=c(.25, .75), na.rm = FALSE)

above <- Q[2]+1.5*iqr

limit<-max(which(test_dist<above)+1)

cell_to_check<-features_rmb_inter$Cluster_THREE[closest$nn.idx[valx,1]]

atoi<-features_rmb_inter$Cluster_THREE[closest$nn.idx[valx,2:limit]]

todos<-all(atoi>=2)

todos_almost<-sum(atoi)>=((length(atoi)*3)-mean(atoi))

if (cell_to_check== 1 && todos) { features_rmb_inter$outlier[closest$nn.idx[valx,1]]<- 0} else {

if (cell_to_check== 1 && todos_almost) { features_rmb_inter$outlier[closest$nn.idx[valx,1]]<- 0} else{

features_rmb_inter$outlier[closest$nn.idx[valx,1]]<- 1}}

}

if (any(features_rmb_inter$outlier==0)){test_out <-0} else {test_out <-1}

features_rmb_inter<-features_rmb_inter[which(features_rmb_inter$outlier==1),]

features_rmb_inter$outlier<-1

}

features_rmb_inter$outlier<-NULL

features<-features_rmb_inter

list_now<-features$order

## # graphical visualization

toberemoved<-setdiff(original_list,list_now)

features$order<-as.numeric(features$order)

x0 <- rmObjects(nmask, toberemoved,reenumerate = F)

sel <- features$order[which(features[,"Cluster_THREE"] !=1)]

x1 <- rmObjects(x0, sel,reenumerate = F)

sel <- features$order[which(features[,"Cluster_THREE"] !=2)]

x2 <- rmObjects(x0, sel,reenumerate = F)

sel <- features$order[which(features[,"Cluster_THREE"] !=3)]

x3 <- rmObjects(x0, sel,reenumerate = F)

res3 = paintObjects(x1, cells, opac=1, col=c("green", "green"), thick=T, closed=T)

res3 = paintObjects(x2, res3, opac=1, col=c('blue', 'blue'), thick=T, closed=T)

res3 = paintObjects(x3, res3, opac=1, col=c('red', 'red'), thick=T, closed=T)

setwd(abb_3class)

imgname=paste(gsub("\\..*","",filename),"_cel_3_class_newcolor.tiff",sep="")

tiff(imgname ,compression='lzw', width = dim(cells)[1], height = dim(cells)[2])

plot(res3)

dev.off()

res3 = paintObjects(x1, allcells, opac=1, col=c("green", "green"), thick=T, closed=T)

res3 = paintObjects(x2, res3, opac=1, col=c('blue', 'blue'), thick=T, closed=T)

res3 = paintObjects(x3, res3, opac=1, col=c('red', 'red'), thick=T, closed=T)

imgname=paste(gsub("\\..*","",filename),"_cel_intercell.tiff",sep="")

tiff(imgname ,compression='lzw', width = dim(cells)[1], height = dim(cells)[2])

plot(res3)

dev.off()

img3 = rgbImage(green=x1, blue=x2,red=x3)

imgname=paste(gsub("\\..*","",filename),"_cel_3_class.tiff",sep="")

tiff(imgname ,compression='lzw', width = dim(img3)[1], height = dim(img3)[2])

plot(img3)

dev.off()

## # clustering 2

data_zw <- features

data_zw$outlier<-NULL

data_zw$m.cy<-NULL

data_zw$m.cx<-NULL

data_zw$order<-NULL

data.scaled <- as.data.frame(apply(data_zw, MARGIN = 2, FUN = scale))

data.scaled.clusters <- kmeans(data.scaled, centers =2, nstart = 25, iter.max = 1000)

data.scaled$cluster <- data.scaled.clusters$cluster

features$Cluster_TWO <-as.factor(data.scaled$cluster)

cluster_1max<-mean(features$diameter_log[features$Cluster_TWO==1])

cluster_2max<-mean(features$diameter_log[features$Cluster_TWO==2])

list_clust<-as.data.frame(rbind(cluster_1max,cluster_2max))

list_clust$id<-c(1,2)

list_clust <-list_clust[with(list_clust, order(list_clust$V1)), ]

list_clust$new_id<-c(1,2)

features$Cluster_1_tochange<-features$Cluster_TWO

features$Cluster_2_tochange<-features$Cluster_TWO

features$Cluster_TWO[features$Cluster_1_tochange==list_clust$id[1]]<-list_clust$new_id[1]

features$Cluster_TWO[features$Cluster_2_tochange==list_clust$id[2]]<-list_clust$new_id[2]

features$Cluster_1_tochange<-NULL

features$Cluster_2_tochange<-NULL

list_now<-features$order

toberemoved<-setdiff(original_list,list_now)

features$order<-as.numeric(features$order)

x0 <- rmObjects(nmask, toberemoved,reenumerate = F)

sel <- features$order[which(features[,"Cluster_TWO"] !=1)]

x1 <- rmObjects(x0, sel,reenumerate = F)

sel <- features$order[which(features[,"Cluster_TWO"] !=2)]

x2 <- rmObjects(nmask, sel,reenumerate = T)

res = paintObjects(x1, cells, opac=1, col=c("green", "green"), thick=T, closed=T)

res = paintObjects(x2, res, opac=1, col=c('blue', 'blue'), thick=T, closed=T)

setwd(abb_2class)

imgname=paste(gsub("\\..*","",filename),"_cel_2_class_newcolor.tiff",sep="")

tiff(imgname ,compression='lzw', width = dim(cells)[1], height = dim(cells)[2])

plot(res)

dev.off()

img2 = rgbImage(green=x1, blue=x2)

imgname=paste(gsub("\\..*","",filename),"_cel_2_class.tiff",sep="")

tiff(imgname ,compression='lzw', width = dim(img2)[1], height = dim(img2)[2])

plot(img2)

dev.off()

features$Cluster_TWO <-as.numeric( features$Cluster_TWO)

## # record of additional information about boundary cells, cell wall, etc.

imagetotalpix=dims[1]*dims[2]

features$imagetotalpix<-imagetotalpix

features$innerpixarea<-innerpixarea

features$cellwallcount<-cellwallcount

intercell<-paintObjects(nmask,inner, col='black', thick=T, closed=T)

intercell<-channel(intercell,"gray")

intercell<-bwlabel(intercell)

intercell<-bwlabel(intercell-allcells)

intercellcount<-sum(intercell, na.rm = TRUE)

features$intercellcount<-intercellcount

features$dataname<-filename

features_boundary$dataname<-filename

## # concatenation all the information of all the images in a single table

if (round==1){DATA_FINAL<<-features} else{DATA_FINAL<<-rbind.data.frame(DATA_FINAL,features)}

if (round==1){DATA_FINAL_boundary<<-features_boundary} else{DATA_FINAL_boundary<<-rbind.data.frame(DATA_FINAL_boundary,features_boundary)}

round<-round+1

} # end from loop

data_f<- na.omit(DATA_FINAL) #remove rows containing NaN

setwd(Table_raw)

write.csv(x=data_f, file="final.csv")

write.csv(x=DATA_FINAL_boundary, file="final_ boundary_information.csv")
